# Individual Health Budgets in Mental Health: Results of Its Implementation in the Friuli Venezia Giulia Region, Italy

**DOI:** 10.3390/ijerph17145017

**Published:** 2020-07-13

**Authors:** Elisa Fontecedro, Morena Furlan, Davide Tossut, Elisabetta Pascolo-Fabrici, Matteo Balestrieri, Luis Salvador-Carulla, Barbara D’Avanzo, Giulio Castelpietra

**Affiliations:** 1 Department of Medicine (DAME), University of Udine, 33100 Udine, Italy; fontecedro.elisa@spes.uniud.it (E.F.); matteo.balestrieri@uniud.it (M.B.); 2 Central Health Directorate, Friuli Venezia Giulia Region, 34100 Trieste, Italy; morena.furlan@regione.fvg.it; 3 Welfare Area, Friuli Venezia Giulia Region, 33057 Palmanova, Italy; davide.tossut@welfare.fvg.it; 4 Clinical Department of Medical, Surgical and Health Sciences, University of Trieste, 34149 Trieste, Italy; elisabetta.pascolofabrici@asugi.sanita.fvg.it; 5 Mental Health Department, WHO Collaborating Centre for Research and Training in Mental Health, Health University Agency of Trieste, 34100 Trieste, Italy; 6 Centre for Mental Health Research, Research School of Population Health, ANU College of Health and Medicine, Australian National University, 2601 Canberra, Australia; luis.salvador-carulla@anu.edu.au; 7 Menzies Centre for Health Policy, Faculty of Medicine and Health, University of Sydney, Charles Perkins Centre, The University of Sydney, 2006 Sydney, Australia; 8 Istituto di Ricerche Farmacologiche Mario Negri, IRCCS, 20156 Milano, Italy; Barbara.Davanzo@marionegri.it

**Keywords:** Individual Health Budget, personal budget, recovery, personalized care, mental disorders, mental health services

## Abstract

Background: Individual Health Budget (IHB) is an intervention for recovery in mental health services, providing personalized care for subjects with severe disorders and complex needs. Little is known on its effectiveness and on the criteria for its delivery. Methods: A total of 67 IHB beneficiaries and 61 comparators were recruited among service users of the Mental Health Department of the Trieste Healthcare Agency, Italy. Data included sociodemographic and clinical variables, type of IHB, and Health of the Nation Outcome Scale (HoNOS) scores. Results: A comparison between groups showed significant differences in several socioeconomic and clinical characteristics. Multivariate logistic regression showed that IHB was positively associated to the 20–49 age group, single status, unemployment, low family support, cohabitation with relatives or friends, diagnosis of personality disorder, and a higher number of hospitalizations. The IHB group was at a higher risk of severe problems related to aggressive or agitated behaviors (OR = 1.4), hallucinations and delusions (OR = 1.5), and impairment in everyday life activities (OR = 2.1). Conclusions: IHB was used in patients with severe clinical and social problems. More resources, however, may be aimed at the working and social axes. More research is needed to better assess clinical and social outcomes of IHB and to adjust their intensity in a longitudinal perspective in order to enhance cost-effectiveness.

## 1. Introduction

Individualized care is a key element in mental health planning and policy [1]. It is linked to recovery-oriented care, which aims to promote positive elements of a personal life in order to ameliorate mental health and well-being [2]. Several approaches may be developed to promote recovery-oriented care in mental health services [3]. To do so, healthcare tools, which can be adapted to different individuals’ needs, should be used. In this context, the personal budget (PB) is an intervention focused on the person’s active participation in the care process. It consists of a sum of money allocated to the person and used for different purposes according to personal needs [4]. PB has been tested in several countries all around the world in the 1990s and assumed a specific meaning and different models according to the cultural and policy context of each country [5]. It is usually delivered to persons with different complex needs, such as chronic illnesses, intellectual disabilities, dementia, substance use, and mental disorders [4,6,7,8,9]. The budget can be delivered through a direct payment to the beneficiary, or through an indirect payment, established and spent by the social or public health care, taking into account the beneficiary’s personal needs [4,9,10]. Some countries have implemented PBs in healthcare and social programs, especially for children (Canada), elderly, and disabled people (the Netherlands, Sweden), but this approach remains in a testing phase [5].

The use of PBs in a mental health setting has been lower than in other fields [7,11], but it is rising due to the adoption of value-based care and the preliminary evidence of multiple positive outcomes for mental health services users [12]. Its effectiveness has been suggested by qualitative studies. Webber et al. [7] underline the positive outcomes associated to personal budget use, emphasizing the potential increase of choice and control experienced by patient with mental health problems: this evidence placed PB in a recovery-oriented perspective, as it focused on individual strengths and preferences, and enables to gain more choice in—and control over—the support for living an autonomous life. Personal narratives from people benefitting from a PB describes a general support in the recovery process [13] and a common perception of being part of the community, which lead to enhanced confidence and skills, new social relationships, and better self-rated mental health [12]. The few studies which investigated the outcomes of PBs for people with mental health problems, showed an increased quality of life and a decrease in inpatient and community mental health service use [7,14,15]. However, most literature on PBs is based on studies from the United Kingdom (UK) and the United States (US) and are qualitative and non-controlled for the great majority [4,7]. Moreover, most of the challenges and criticisms related to PBs in the context of mental health are based on the UK [15,16], which is also the leading proponent of this approach. Issue regarded, for instance, going beyond a resource allocating system focused on services, the increased bureaucracy, and the difficulties in managing PB founding among users and services, including the risk of a long-term dependency [5]. Although literature on the use and outcome evaluation of PB in Italy is scarce [17,18,19], first PB programs started as early as the 1990s in connection with the deinstitutionalization of psychiatric hospitals and the construction of a new model of psychiatric rehabilitation [17,20,21]. Nonetheless, only few Italian regions have adopted the PB as an integration tool for social and health interventions [22]. The Italian version of PB, called “Individual Health Budget” (IHB), has been defined as “the complex of economic, professional, and human resources needed to trigger a process aimed to restoring a person to an acceptable level of social functioning, through an individual rehabilitation process” [17]. IHB programs implemented in Italian regions are in any case differentiated by features directly regulated at the level of regional policy [21,22].

In this framework, the Friuli Venezia Giulia (FVG) Region has been the first in Italy to use the IHB in a mental health context, following the approval of a regional law in 2006 [22]. However, the Mental Health Department of Trieste has been already experimenting with IHB, following the spirit of the Italian psychiatric reform, based on an holistic approach combining healthcare and welfare systems working together to allow users regaining civil and social rights [23]. Originally, IHBs were used in Trieste as a mean for reallocating resources earmarked for residential care. Afterwards, this stimulated the development of individual plans starting from personal needs addressed initially to different housing solutions, then expanding to other determinants of health, such as working and social relationships [20]. This model was then replicated all over FVG in line with the principles of the reform [24].

Despite the growing use of IHB in Italian mental health services, however, scientific evidences on IHB beneficiaries’ characteristics and IHB outcome have to be better assessed. Furthermore, there is still no consensus, nor shared guidelines, on the criteria of access to the IHB and on the features to consider in order to clearly define the attribution of IHB intensity. In particular, more research is needed to better identify to which extent clinical or socioeconomic characteristics may influence the delivery of an IHB to patients with mental health disorders [25]. A better knowledge of IHB beneficiaries’ characteristics may help to assess whether IHB is applied according to regional health policy, and, consequently, to better drive tailored interventions. Furthermore, the use of validated instruments to evaluate a health budget’s outcome, also in a longitudinal long-term perspective, is highly needed [25].

The aims of this study are (a) to describe the use of the IHB in a mental health setting comparing the socioeconomic and clinical characteristics of the beneficiaries of an IHB with those of subjects who are not; and (b) to compare the psychosocial features in beneficiaries and not beneficiaries of an IHB using a validated scale.

## 2. Materials and Methods

This comparative cross-sectional study evaluated the IHB implementation in the mental health setting of the FVG Region. It is part of a multicenter observational cross-sectional study promoted by the Central Health Directorate of the FVG Region in collaboration with the Welfare Area of the FVG Region, which deals with the need of the regional government to assess the outcome of the financed Individualized Therapeutic Rehabilitation Plans (ITRPs), addressed to patients with severe mental disorders.

All participants were treated in accordance with the WMA Declaration of Helsinki. The study was approved by the ethics committee of the FVG Region.

### 2.1. Description of the Field

Data was collected in the Mental Health Department (MHD) of the Health University Agency of Trieste. Trieste has been the leading center of mental health reform in Italy, with a later expansion to other southern European countries [26]. From the first closure of the mental hospital in 1980, Trieste has built an original mental health model made up of open door–no restraint services and very low use of compulsory interventions, which is a benchmark in comparison with other realities [23,27]. The MHD of Trieste coordinates a wide range of services and facilities such as the Community Mental Health Centers (CMHCs), the general hospital psychiatric unit (GHPU), and the supported housing facilities and daily centers. The main services of the MHD are four CMHCs, each looking after a catchment area of 50,000 to 65,000 inhabitants. All CMHCs in Trieste area are open 24 h a day, 7 days a week, with four to eight beds each. CMHCs directly deal with a full range of psychiatric needs, including management of acute conditions, prevention of mental disorders, treatment, and rehabilitation, which includes finding a job and a home [23,24].

### 2.2. The Individual Health Budget (IHB)

An IHB is a resource of the MHD linked to an ITRP. The ITRP focuses on people with mental disability and their needs, defining objectives, modalities, procedures, and resources. It is time-limited and subjected to planned revision and outcome assessment. The IHB is the financial component of the ITRP and it is proportionated to its needs. IHB is an investment that the public system makes on the person through the intermediary operating in the private sector (social cooperatives) in order to create conditions to reactivate the social functioning and concrete opportunities of living, working, and socializing.

Each IHB is defined in terms of intensity which is established according to the different and specific needs of each patient. The IHB is divided into three axes (living, working, social relationship). Interventions are identified for each axis to provide a rehabilitative response to be adapted to the personal needs. These interventions are calculated in economic terms in order to establish the share of resources which should be invested in each of the axes. The economic value of each intervention is calculated taking into account the complex of the cost items that make up the intervention itself (professionals’ costs per hour plus meals, rents, transport, food, utilities, materials, equipment, etc.). The intensity of IHB is, thus, grouped into 8 categories according to the severity degree of the psychopathological conditions, the level of functional impairment, the rehabilitative needs, and the duration of intervention identified by the CMCHs’ team assessments: High/Out of Threshold (when the necessary needs and resources exceed the High threshold), High, Medium-High, Medium, Medium-Low, Low, according to the specification defined by the FVG Health Authority [28]. As a consequence, the interventions in each axis (housing, working, and social relationships) are grouped using the same categories: High, Medium-High, Medium, Medium-Low, and Low.

Usually the beneficiaries of an IHB have complex needs, which do not involve only the severity of a psychiatric illness, but also social and cultural elements. As a consequence, this requires a set of individualized interventions. Beneficiaries of an IHB usually are

1. People with serious impairment in personal and social functioning, in a post-acute phase of their illness;
2. People with moderate to severe impairment in personal and social functioning, in the long-term phase of their illness;
3. People with different degrees of autonomy and impairment in personal and social functioning.

### 2.3. Description of the Sample

Participants were recruited from the 4 CMHCs of Trieste from 1 January 2019 to 31 March 2020. All subjects were regularly followed by their CMHCs for a severe mental disorder. They were divided into two groups: a first group of patients benefitting from an IHB, and a second group of “comparators”, whose care was maintained “as usual”. The IHB sample included subjects who were benefitting from an IHB on 31 December 2018.

The extraction of participants was applied using a randomized procedure firstly to all IHB beneficiaries belonging to the 5 MHDs of the region through 3 variables: age group (0–24; 25–49; ≥50), sex, and intensity of IHB. We obtained 109 clusters from which 64.64% were extracted. Secondly, the 67 subjects belonging to the MHD of Trieste were selected as “IHB group” for the present study. Thirdly, 67 subjects of the “comparator” group from Trieste MHD patients were extracted using a randomized procedure, based on sex and the same age groups, within all subjects who were not benefitting from an IHB on 31 December 2018. Six subjects were excluded from the group due to the inability of getting in touch with them. Consequently, the final comparator group resulted of 61 subjects. All personal data (first name, last name, date of birth) were converted into an anonymous unique code in accordance with the Data Protection Act (EU Regulation 679/2016).

### 2.4. Data Collection

Data were collected through a questionnaire. Questionnaires were distributed by the research team to the patient’s referral professional, who could be either the case manager or the psychiatrist, who filled it. Case managers are usually the frontline professionals (in most cases nurses), who are working daily with patients in the community on several care aspects, such as medication delivery and medication compliance, outpatient services activities, home visits, housing, employment, and social support. The referral professionals collected all available patient information by direct interviews with them or by additional information retrieved from family members and case meetings, or by consulting clinical diaries.

### 2.5. Description of the Questionnaire

The questionnaire was divided into two parts.

The first part investigated:

1. Sociodemographic items: age groups, sex, marital status (single, married, divorced, widower), citizenship, level of education related to years of study (≤5, 6–8, 9–13, and ≥14);
2. Economic items: occupational status, disability benefit, and/or other economic benefits;
3. Family contest: cohabitation, detected difficulties in family support;
4. Clinical items: date of first contact with CMHC, diagnosis according to ICD-10 which was then divided into 5 categories: psychoactive substances disorders (codes F10–F19), psychotic disorders (codes F20–F29), affective disorders (codes F30–F49), personality disorders (codes F60–F69), other psychiatric disorders (codes F50–F59), number and length of hospitalizations, both voluntary and compulsory, in the 365 days prior to the activation of the IHB.

The second part took into account the items of the IHB project:

1. IHB intensity (High/Out of Threshold, High, Medium-High, Medium, Medium-Low, Low);
2. Workplace: training activities, supported working, not supported working;
3. Work axis intensity: High, Medium-High, Medium, Medium-Low, Low;
4. Living setting:
  - Therapeutic-rehabilitative dwellings, addressed to people with severe mental health problems and no supportive family network (on 12 or 24 h);
  - Transitional flats (on 12 or 24 h) used as temporary housing solutions;
  - Supported housing (on 12 or 24 h) which is personalized support at people’s own homes, with varying degrees of intensity;
5. Living axis intensity: High, Medium-High, Medium, Medium-Low, and Low;
6. Social axis intensity: defined according to the level of severity of relational problems (High, Medium-High, Medium, Medium-Low, and Low).

### 2.6. Description of Health of the Nation Outcome Scale (HoNOS)

The outcome assessment was carried out by administering the Italian version of the Health of the Nation Outcome Scale (HoNOS) [29]. HoNOS is a multidimensional assessment scale which detects both clinical and psychosocial problems, regardless of the diagnostic category [29]. It is made up of 12 items that evaluate the extent of the problem raised in the previous 2 weeks. Each item is evaluated on a score scale that ranges from 0 (no problem) to 4 (maximum severity of the problem). A total score can be obtained from the sum of each item’s scores and a 4-level severity index (subclinical, mild, moderately severe, and very severe) by combining responses to the various items.

All professionals who evaluated the sample’s participants, received a comprehensive training on HoNOS, organized by the MHD of Trieste.

### 2.7. Data Analysis

Numerical variables were summarized using mean and standard deviation (SD) as measures of central tendency, while ranges were used as measures of dispersion. Dichotomous categorical variables were tabulated into a contingency table and the chi-square statistics (*χ*^2^) were used to test the differences between observed and expected frequencies. Means were compared by a “Welch Two Sample *T*-test”, which is an adaptation of a *T*-test for samples that have unequal sample sizes. Moreover, OneHot encoding of dichotomous or categorical variables was performed to allow separate regression coefficients to be matched for each possible value of the discrete variable, making a more precise regression model [30]. A couple of learning algorithms (decision trees, logistic regression, and Support Vector Machines) were tested to assess the association between outcomes and predictors (socio-demographic and clinical variables; HoNOS items). Multivariate logistic regression achieved the best results for the entire set of predictors (108 dummy coded variables), with an accuracy of 0.75. We also performed a feature selection algorithm to improve accuracy, avoiding overfitting and simplifying the model [31]. This reduced the set of dummy coded variables to 60, excluding dummy variables which resulted as not significant for the regression model. A bootstrap with 2000 repetitions was performed and an accuracy of 81.9% with a 95% Confidence Interval (95% C.I.) of 71.8–92.3 was achieved. Odds ratio (OR) with relative Wald 95% C.I. was estimated for each of the selected variables. A *p*-value (*p*) of <0.05 was set as the threshold for statistical significance. Descriptive and inferential analyses were performed using the statistical software R (RStudio, Boston, MA, United States) and Python (Python Software Foundation, Wilmington, DE, United States).

## 3. Results

### 3.1. Sociodemographic and Clinical Features

As shown in Table 1, the majority of subjects included in the study were males (58.6%). Ninety-four percent were Italians. The fifty to fifty-nine years old age group was the most represented. More than 70% were unmarried. More than 60% of subjects were unemployed, albeit this was more likely in the IHB group compared to the comparators (*χ*^2^ (*p*) < 0.005). Subjects in the IHB group were also more likely to benefit from a disability check (*χ*^2^ (*p*) < 0.001). The pattern of cohabitation was also significantly different between the IHB and comparator groups (*χ*^2^ (*p*) < 0.001), with IHB more likely to live with relatives and friends. However, 30% of subjects in both groups were supported by families and only six subjects (5%) were living in residential/community facilities.

Psychotic disorders were the main diagnosis in both samples representing more than 80% of the overall sample. The mean number of hospitalization during the study period was 0.9. Voluntary hospitalizations were highly prevalent (97.4%). The mean length of hospitalization was 20.4 days (range = 1–305 days). Voluntary hospitalization differed significantly in number and length of hospitalization in the IHB group compared to comparators (Table 1).

### 3.2. IHB Variables

**Figure 1:**
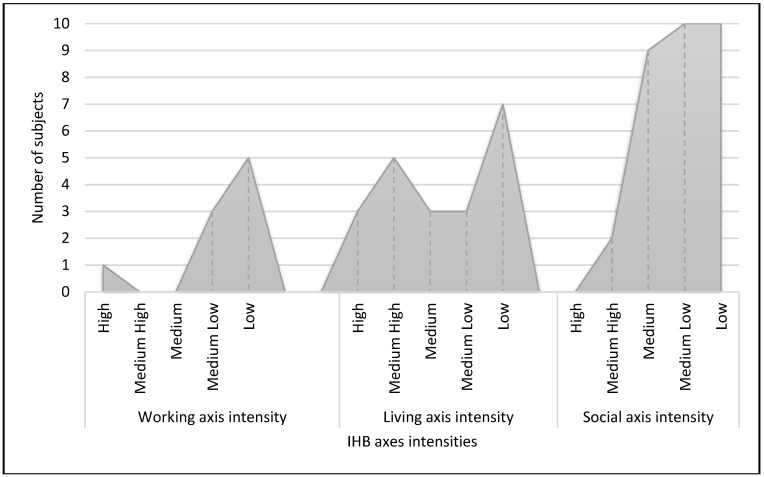
Distribution of the number of subjects in the Individual Health Budget (IHB) group according to working, living, and social axes intensity.

A medium-high to medium-low intensity was the most used IHB (*N* = 42; 62.7%), while only three subjects benefitted from high intensity IHB and six of IHB out of threshold. As shown in Figure 1, this estimate was similar in working and social axes intensity, while subjects were more fairly distributed among living axis intensity. When subjects benefitted from an IHB on the living axis (*N* = 43; 64.2%), the most used living settings were transitional flats (*N* = 19, 44.2%) and supported housing (*N* = 21; 48.8%). Only nine patients (13.4%) benefitted from the IHB covering working axis, with training activities (*N* = 2; 22%) or tutored working (*N* = 7, 73%).

### 3.3. HoNOS Variables

As shown in Table 2, cognitive problems, problems related to hallucination and delusions, in the activities of everyday life and in the availability of resources for work and recreation activities differed significantly between the IHB and comparator groups (*T*-test (*p*) < 0.01). Neither the mean scores used to identify clinical severity nor the mean total score differed significantly between the two groups (*T*-test (*p*) = 0.23).

### 3.4. Multivariate Logistic Regression Analyses

The multivariate logistic regression analyses showed that the IHB group was positively associated with an age between 20 and 49 years, a single marital status, a cohabitation with relatives or friends, and a disability check up to 100%, when compared to the comparator group. IHB beneficiaries were also more likely to have a diagnosis of personality disorder, a higher number of voluntary hospitalizations, and a longer length of compulsory hospitalizations (Table 3). In contrast, an age of >70, no difficulties in family support, and a diagnosis of psychotic disorder were inversely associated to IHB (Table 3).

## 4. Discussion

Our study highlighted that IHB beneficiaries were generally males, aged between 40 and 59 years, singles, economically inactive with a disability check, and diagnosed with psychotic disorders. They were also at a higher risk of having moderately severe problems with regard to cognitive and physical impairment, hallucinations and delusions, and activities in everyday life. Nonetheless, the clinical and functional impairment, measured with the HoNOS total scores, did not differ between IHB and comparators.

Although gender and age of IHB beneficiaries were in line with previous Italian studies [17,19], the 20–29 and 40–49 years old age groups were positively associated with IHB allocation. This confirmed previous findings that considered IHB a flexible tool, which can be used at different ages, depending on an individual’s needs [32]. Nonetheless, IHB in the elderly was rarely used, as indicated in other studies [7]. In line with Forder et al. [14], IHB subjects were more likely to live with relatives or friends. However, this seems to differ from the general features of Mediterranean countries, where patients are more likely to live inside or closely connected to families compared to Anglo-Saxon countries [33]. The absence of a proximal family network, as partner or parents, was also confirmed by the fact that more than 30% of data on family support was missing in this group, which may be explained by the fact that the living environment was shared with other people like friends or relatives. Albeit previous studies were focused more on active and affective family attitude, which can encourage and motivate patients to treatment, prevent isolation, and help them face the disabilities of the illness [34]. This can be extended to all supporting relationships, which can play a key role in the recovery process [35]. Although living alone should not be seen only in a social deprivation perspective, the presence of a strong social support network may be a significant predictor of mental health service use [33]. Nonetheless, the fact that most IHB beneficiaries did not live alone can be explained by the great use of supported accommodations in our sample, underlying the importance of supported housing solution availability in the specific catchment area of Trieste [20]. Moreover, more than 70% of IHB beneficiaries were psychotic and economically inactive, which was paralleled by 60% of them receiving a disability check. This was consistent with previous findings from the FVG Region, which indicated that psychotic disorders were significantly associated with being economically inactive [36]. Severe mental illness can highly impair work capacity and is also associated with high unemployment rates [36,37,38,39,40,41,42]. Further, economic insecurity is common among patients with severe mental disorders [43,44], as well as functional impairment, which prevents them from living an independent life [45]. Despite this evidence, working interventions, which can highly enhance functional impairment [46], have not been widely used in the IHB group. The most of IHBs were indeed focused on the living axis [47], which comprehended a variety of “housing opportunities”, such as therapeutic–rehabilitative dwellings, individual and grouped flats, and personalized support at home. This has allowed a reduction of psychiatric staffed facilities use over the years in FVG [48]. Housing is a key issue for the recovery process of people with mental health illness [35], and it has been also identified as an indicator of social functioning [49]. IHB can support sustainable independent living with various approaches, with the goal of decreasing residential solution and institutionalization [20].

In relation to other clinical variables, we highlighted that the risk of hospitalization in the IHB sample compared to comparators was 1.4-fold higher, regardless of diagnosis, which reflected the complex clinical condition of these subjects. This complexity was confirmed by a higher mean HoNOS total score in the IHB group, even not statistically significant, while it was highly significant in specific HoNOS items, like hallucinations and delusions, hyperactive, aggressive, destructive or agitated behaviors, as well as in cognitive problems. Cognitive impairment can be also considered as a main driver in affecting patients’ occupational, social, and economic functioning [50]. Furthermore, subjects in the IHB group were found at a two-fold increased risk of impairment in basic activities of daily living, such as washing, dressing, cooking, money management, or household shopping. This was consistent with previous studies, where people benefitting from a personal budget were found to be significantly more likely to be unable to perform a number of daily living activities compared to the control group [14].

To date, our findings suggested that IHB beneficiaries presented a condition of significant clinical attention, where the integrated and personalized intervention, which is the core of IHB, should be crucial and more fostered. IHB has the potential of enhancing health of beneficiaries by modulating specific interventions according to their needs [5] in order to avoid unplanned use of inpatient and crisis care services [15]. Findings from the UK observed that PB holders used less national health care services, including inpatient care, than those not using it [14]. Indeed, our findings indicated the opposite. This discrepancy may be related to the different approaches in PB allocation in Italy and the UK [4,19]. Nonetheless, our study may indicate that more efforts should be aimed at rehabilitation interventions especially on daily living for IHB beneficiaries, with the goal of improving their clinical condition. On the other hand, personalized planning offered by IHB itself may be a potential answer to these issues, since they focus primarily on a patient’s life context and needs [5,48]. In our study, in fact, we found lower ratings in the IHB sample with regard to problems related to occupation and activities. This item took into account accessibility, availability, and suitability of the occupational and recreational environment provided to the patient, which was, thus, enhanced by IHB. In contrast, mild problems in social relationships were found to be associated with IHB. This finding, associated to a moderate effort in the social axis, suggested that social impairment is not crucial for the allocation of IHB. This might be linked to the complexity of a social dimension, which is influenced not only by the severity of psychopathological symptoms, but also by other factors, such as setting, employment, and partnership [51]. Targeted interventions addressed to encourage access to employment might influence social integration by improving communication and rebuilding a new social network in the work setting [52]. Moreover, additional tools, developed to enhance users’ social skills, like peer support, can be integrated with IHB [53,54]. Few studies, however, investigated the relationship between mental illness, patient characteristics, and budget outcomes in the different axes. One study, for instance, investigated predictors of employment related to placement budgets, concluding that a better global functioning is associated with finding and keeping a job [55].

In any case, IHB can be considered a flexible tool that may help beneficiaries to take control of their recovery, regardless of illness phase and diagnosis, dealing with the most severe conditions with the heaviest burden of care [20]. This was also confirmed by previous Italian studies, which highlighted a general improvement in several HoNOS items in IHB beneficiaries with longitudinal evaluations. However, these studies were based on a limited sample size, and they did not provide a comparison with comparators [17,18,19].

## 5. Strengths and Limitations

To our knowledge, this is the first Italian study analyzing socioeconomic and clinical factors in patients with mental disorders related to IHB allocation, comparing them to comparators. This method has been rarely used and mainly in non-psychiatric samples [14]. Another strength was that it analyzed IHB, which acts on different clinical and social dimensions, among patients in a real-world mental care setting. This kind of research can represent an opportunity to highlight factors impacting on IHB implementation, as well as enhancing the development of implementation theories on this subject [56]. Further, the use of HoNOS enabled a preliminary outcome evaluation in a routinely clinical context, taking into account every source of information about the patient with an adequate coverage of clinical and social function [29,57].

This study has a number of limitations. Firstly, the design was cross-sectional, and no longitudinal analysis has been performed at the moment. This is a necessary step in order to evaluate IHB in terms of patient improvement over time, both from a clinical and a psychosocial perspective, shedding a light on the growing of an individual’s independence and ability to self-manage the process of care [19]. Secondly, HoNOS was evaluated by mental health professionals, in particular case managers. This may have led to a recall bias with a consequent overestimation of the severity of IHB problems by assigning higher scores compared to comparators. International evidence suggested that HoNOS ratings were less reliable when completed by clinicians, rather than research staff [29]. Furthermore, HoNOS provides a problem-centered evaluation, excluding functioning features, which may be better defined by other tools, such as the Global Assessment of Functioning scale [18]. Next research steps, thus, may take into account the possibility of using other scales to enhance the outcome assessment. Thirdly, several missing data in the family support pattern may have lowered the significance of this variable, which would have better explained social outcomes. Fourthly, the small sample size hindered a detailed analysis in determining subgroups. This was the case of personality disorders, for instance, which were positively associated to IHB, but they were representing only 7.7% of the whole sample. In contrast, this could have also affected the inverse association between psychotic disorders and IHB allocation, since the difference between IHB and comparators consisted only in six subjects. Nonetheless, the diverse association between these clinical diagnoses and IHB is not easy to explain. Both disorders, in fact, are associated with functional impairment, unemployment, and interpersonal deficits [45,46]. Fifthly, we did not include pharmacological assessment, which could have affected HoNOS ratings. In mental health services there are patients with little or no compliance to pharmacological therapy [58]. Since we could not assess the distribution of patients following a regular treatment in the two groups, this could have confounded our results, leading to higher HoNOS ratings, due to scarce compliance, in a group compared to another. Finally, we had no qualitative data on how IHB is experienced by users. This would also be of help in the future for enhancing the whole frame of IHB use in a mental health setting. However, previous research from Italy indicated a high customer satisfaction in relation to IHB allocation [17,18].

## 6. Conclusions

This study has identified clinical and socioeconomic features of people with mental disorders benefitting from an IHB intervention, highlighting the items which mainly drive IHB delivery. In general, IHB was provided to patients with a more severe clinical condition and social problems in some areas, such as daily life activities. This seems adequate since IHB is primarily focused on patients with multiple disabilities, both from a clinical and social perspective [19]. They were also more likely to live with relatives or friends, rather than proximal family, which may indicate a greater use of different housing opportunities. Moreover, IHB was mainly focused on the living axis, while fewer resources were orientated around employment and social activities. This highlighted the need of increasing the resources addressing working and social axes [23], possibly targeted at different gender and age groups. Moreover, a greater involvement of the users in the co-production of the IHB program may further enhance personal empowerment and well-being [19]. Further research is needed, however, to better assess the IHB intensity on an individualized basis, according to the clinical and social profile. It is further striking to evaluate clinical and social outcomes longitudinally in order to establish the effectiveness of IHBs. This is also crucial on a cost-effective perspective, since identifying predictors of a IHB’s effectiveness may greatly enhance the allocative efficiency by informing decisions on resource allocation [14].

## Figures and Tables

**Table 1:** Sociodemographic and clinical characteristics of the Individual Health Budget (IHB) and comparator group. Significant *p*-values at the *χ*^2^ and *T*-test are highlighted in bold.

	IHB Group (*n* = 67)	C Group (*n* = 61)	
	*N* (%)	*N* (%)	*χ*^2^ (*p*)
**Gender**			
Female	25 (37.3)	28 (45.9)	0.389
Male	42 (62.7)	33 (54.1)	
**Age Group**			
20–29	12 (17.9)	3 (4.9)	0.136
30–39	8 (11.9)	9 (14.7)	
40–49	16 (23.9)	9 (14.7)	
50–59	14 (20.9)	19 (31.1)	
60–69	12 (17.9)	12 (19.7)	
≥70	5 (7.5)	9 (14.7)	
**Nationality**			
Italian	65 (97)	56 (91.8)	0.161
Other	2 (3.0)	5 (8.2)	
**Marital Status**			
Single/Never married	55 (82)	43 (70.5)	0.428
Widower	1 (1.5)	1 (1.7)	
Married	2 (3.0)	4 (6.5)	
Separated/Divorced	4 (6.0)	8 (13.1)	
Unknown	5 (7.5)	5 (8.2)	
**Educational Level (Years)**			
≤5	1 (1.5)	2 (3.3)	0.067
6–8	46 (68.6)	32 (52.4)	
9–13	12 (17.9)	21 (34.4)	
≥14	2 (3.0)	5 (8.2)	
Unknown	6 (9.0)	1 (1.7)	
**Occupational Status**			
Employed	6 (9)	16 (26.2)	**0.009**
Looking for a job	5 (7.5)	4 (6.5)	
Internship	5 (7.5)	1 (1.7)	
Economically inactive	47 (70.0)	33 (54.1)	
Unknown	4 (6.0)	7 (11.5)	
**Cohabitation with**			
Alone	21 (31.3)	24 (39.3)	**<0.001**
Parents	8 (11.9)	19 (31.1)	
Partner	2 (3.0)	6 (9.8)	
Relatives/Friends	23 (34.3)	4 (6.6)	
Residential facilities	2 (3.0)	4 (6.6)	
Other	11 (16.5)	4 (6.6)	
**Difficulties in Family Support**			
None	15 (22.4)	23 (37.7)	0.58
Minor	12 (17.9)	13 (21.4)	
Mild	3 (4.5)	6 (9.8)	
Moderate	8 (11.9)	8 (13.1)	
Severe	6 (9.0)	3 (4.9)	
Unknown	23 (34.3)	8 (13.1)	
**Disability Check**			
0	22 (40.0)	41 (67)	**<0.001**
>0 and <100	2 (4.0)	10 (16.5)	
100	31 (56)	10 (16.5)	
**Psychiatric Diagnosis**			
Psychoactive substances disorders	1 (1.5)	0	0.119
Psychotic disorders	49 (73.0)	55 (90.1)	
Affective disorders	6 (9.0)	4 (6.5)	
Personality disorders	5 (7.5)	1 (1.7)	
Other psychiatric	6 (9.0)	1 (1.7)	
	**Mean (SD)**	**Mean (SD)**	***T*-test (*p*)**
**Number of Hospitalization**			
Voluntary	1.34 (3.16)	0.36 (0.91)	**0.017**
Compulsory	0.03 (0.17)	0.02 (0.13)	0.614
**Length of Hospitalization (Days)**			
Voluntary	20.42 (40.77)	19.54 (60.08)	**0.021**
Compulsory	0.36 (2.06)	0.11 (0.90)	0.469

IHB: Individual Health Budget; C: comparator; *N*: number; SD: standard deviation.

**Table 2:** Mean and standard deviations (SD) of scores according to Health of Nation Outcome Scale (HoNOS) items in the Individual Health Budget (IHB) and comparator groups. Significant *p*-values at the *T*-test are highlighted in bold.

	IHB Group (*n* = 67)		C Group (*n* = 61)		
HoNOS Items	Mean	SD	Mean	SD	*T*-Test (*p*)
1. Hyperactive, aggressive, destructive, or agitated behaviors	0.67	1.05	0.52	0.75	0.34
2. Deliberately self-harming behavior	0.09	0.38	0.14	0.43	0.53
3. Problems related to alcohol or drug use	0.48	0.94	0.54	1.07	0.72
4. Cognitive problems	1.58	1.18	1.02	1.24	**0.01**
5. Problems arising from somatic disease or physical disability	1.01	1.24	1.20	1.05	0.36
6. Problems related to hallucinations and delusions	1.58	1.36	0.91	1.16	**<0.01**
7. Problems related to depressed mood	0.64	0.90	0.95	0.87	**0.05**
8. Other mental and behavioral problems	1.80	1.18	1.43	1.24	0.10
9. Relational problems	1.99	1.15	2.05	1.31	0.76
10. Problems in the activities of everyday life	2.39	1.09	1.53	1.44	**<0.01**
11. Problems in living conditions	1.02	1.17	1.13	1.19	0.57
12. Problems in the availability of resources for work and recreation activities	0.94	1.09	1.69	1.28	**<0.01**
**HoNOS Output**					
Subclinical (0 item >2)	3.40	3.29	2.38	2.77	0.58
Mild (≥1 item =2)	8.86	3.80	8.07	4.62	0.62
Moderately Severe (1 item >3)	9.18	3.34	10.31	3.17	0.41
Very Severe ((≥2 item ≥3)	19.05	5.89	19.52	6.03	0.76
Total score	14.13	7.51	12.49	7.91	0.23

IHB: Individual Health Budget group; C: comparators; SD: standard deviation.

**Table 3:** Association between sociodemographic and clinical variables, and Health of the Nation Outcome Scale (HoNOS) items in relation to the allocation of Individual Health Budget (IHB). Only variables identified with a feature selection algorithm (*N* = 60) were considered in the analysis. Odds ratios (OR) and 95% confidence intervals (95% C.I.) were estimated by means of multivariate logistic regression analysis. The Individual Health Budget group (IHB) was used as reference category (OR = 1.0). Significant ORs are highlighted in bold.

Sociodemographic and Clinical Variables	Multivariate Logistic Regression	
	OR	95% C.I.
**Age Group**		
20–29	**1.74**	**1.23–2.46**
40–49	**1.88**	**1.15–3.08**
50–59	0.73	0.50–1.07
≥70	**0.57**	**0.40–0.81**
**Marital Status**		
Single/Never married	**1.52**	**1.05–2.19**
Separated/Divorced	**0.61**	**0.44–0.84**
**Education Level (Years)**		
6–8	1.09	0.75–1.59
9–13	0.73	0.50–1.08
**Occupational Status**		
Looking for a job	1.06	0.62–1.79
Internship	1.23	0.93–1.63
Economically inactive	1.31	0.85–2.00
**Cohabitation with**		
Alone	0.78	0.51–1.19
Parents	0.40	0.27–0.59
Relatives/Friends	**2.52**	**1.67–3.82**
**Difficulties in Family Support**		
None	**0.53**	**0.35–0.80**
Minor	0.76	0.41–1.40
**Diagnosis**		
Psychotic disorders	**0.49**	**0.35–0.69**
Affective disorders	1.06	0.77–1.45
Personality disorders	**1.24**	**1.04–1.48**
**Disability Check ^a^**	**2.79**	**1.87–4.17**
**Number of Hospitalization**		
Voluntary	**1.42**	**1.12–1.79**
**Length of Hospitalization (Days)**		
Compulsory	1.06	0.69–1.61
**HoNOS Items**		
**1. ** **Hyperactive, Aggressive, Destructive, or Agitated Behaviors**		
2 Mild problem	**0.56**	**0.38–0.83**
4 Severe to very severe problem	**1.40**	**1.06–1.86**
**2. ** **Deliberately Self-Harming Behavior**		
0 No problem	**2.00**	**1.42–2.82**
2 Mild problem	0.84	0.64–1.09
**3. ** **Problems Related to Alcohol or Drug Use**		
0 No problem	1.41	0.96–2.07
1 Minor problem	0.77	0.52–1.16
**4. ** **Cognitive Problems**		
0 No problem	0.78	0.50–1.21
1 Minor problem	0.99	0.64–1.53
3 Moderately severe problem	**1.66**	**1.14–2.43**
4 Severe to very severe problem	0.84	0.62–1.13
**5. ** **Problems Arising from Somatic Disease or Physical Disability**		
0 No problem	**2.35**	**1.54–3.58**
3 Moderately severe problem	**1.75**	**1.22–2.50**
**6. ** **Problems Related to Hallucinations and Delusions**		
0 No problem	**0.48**	**0.34–0.68**
1 Minor problem	**0.59**	**0.39–0.88**
2 Mild problem	**2.26**	**1.60–3.19**
3 Moderately severe problem	1.22	0.82–1.82
4 Severe to very severe problem	**1.52**	**1.12–2.07**
**7. ** **Problems Related to Depressed Mood**		
0 No problem	**1.76**	**1.13–2.74**
**8. ** **Other Mental and Behavioral Problems**		
0 No problem	1.38	0.93–2.05
2 Mild problem	**1.66**	**1.08–2.57**
4 Severe to very severe problem	1.25	0.83–1.97
**9. ** **Relational Problems**		
1 Minor problem	0.81	0.58–1.14
2 Mild problem	**1.64**	**1.06–2.54**
3 Moderately severe problem	1.38	0.94–2.03
**10. ** **Problems in the Activities of Everyday Life**		
0 No problem	**0.36**	**0.25–0.52**
2 Mild problem	1.45	0.94–2.23
3 Moderately severe problem	**2.10**	**1.43–3.08**
**11. ** **Problems in Living Conditions**		
0 No problem	1.32	0.87–3.01
2 Mild problem	0.73	0.47–1.14
4 Severe to very severe problem	0.81	0.62–1.05
**12. ** **Problems in the Availability of Resources for Work and Recreation Activities**		
0 No problem	**2.09**	**1.40–3.14**
1 Minor problem	1.33	0.86–2.06
3 Moderately severe problem	0.71	0.46–1.10
4 Severe to very severe problem	**0.51**	**0.33–0.78**
**HoNOS Output**		
Mild (≥1 item =2)	**0.59**	**0.39–0.90**
Moderately severe (1 item >3)	1.15	0.77–1.71

^a^ Treated as continuous variable (from 0 to 100).
